# Collaboration Between Humans and Robots in Organizations: A Macroergonomic, Emotional, and Spiritual Approach

**DOI:** 10.3389/fpsyg.2022.855768

**Published:** 2022-05-19

**Authors:** Violeta Firescu, Mirabela-Luciana Gaşpar, Ioan Crucianu, Eliade Rotariu

**Affiliations:** ^1^Management Research Center for Organizational Sustainability, Department of Management and Economic Engineering, Technical University of Cluj-Napoca, Cluj-Napoca, Romania; ^2^Department of Design Engineering and Robotics, Technical University of Cluj-Napoca, Cluj-Napoca, Romania; ^3^Faculty of Orthodox Theology, Institute for Doctoral Studies, Babeş-Bolyai University, Cluj-Napoca, Romania; ^4^De KLAUSEN, Cluj-Napoca, Romania

**Keywords:** organizational ergonomics, management systems, employees’ identity awareness, human capital, adaptability to change, new technologies

## Abstract

The new managerial challenges are related to finding solutions for complex problems, inside some more and more complex management systems, in a continuously changing organizational context. Competitivity and progress imply a continuous positive change and the need to accept, respond, and adapt to the organization’s internal and external environments changes. This brief research report aims to point out the organizational ergonomics’ contribution to employees’ wellbeing through a systemic, emotional, and spiritual approach to man’s interaction with technology, systems, and organizational environment. The research methods used were the multidisciplinary bibliographic study and the interview. Three semi-structured interviews were taken to explore today’s challenges and new 4.0 technologies’ impact, especially robots, on the company and on employees’ wellbeing and spiritual fulfillment. The novelty comes from the analysis of new technologies’ impact on the human factor from the spiritual point of view. Our main results have to do with the shaping of a model for human capital’s valorization and with suggesting a list for monitoring human capital valorization in the company. This article’s main conclusion shows that the organizations’ management must be prepared to manage future challenges by improving the employee’s abilities, adaptability to change, and collaboration with robots.

## Introduction

The new challenges inside organizations are given by the Industry 4.0 concept implementation (Bortolini et al., 2017; Mikulic and Stefanic, 2018; Kadir et al., 2019; Ghobakhloo, 2020; Gualtieri et al., 2020; Neumann et al., 2021; Reiman et al., 2021; Ching et al., 2022) and by a continuous change in the customers’ requirements related to products and services characteristics (Bortolini et al., 2017; Bolis et al., 2020).

The advantages, opportunities, and benefits of Industry 4.0 such as (Oztemel and Gursev, 2020): high productivity, improved innovation capability, improved flexibility with decreased costs, customized products, etc., cannot be disputed. Robotic technology use gained ground in industrial manufacturing processes (Kadir et al., 2019; Reiman et al., 2021; Ching et al., 2022), assembly systems (Bortolini et al., 2017; Gualtieri et al., 2020; Weckenborg et al., 2022), logistics and retail operation (Bertacchini et al., 2017), and in everyday life (Sanders et al., 2019), robots becoming “key players” (Oztemel and Gursev, 2020) in the various domain: industry, logistics, retail, education, surgery, procurement, etc.

Considering the multiple facets of the Industry 4.0 concept [such as digitalization, Internet of Things (IoT), cloud-based systems, artificial intelligence, big data analytics, exoskeletons, and collaborative and cooperative robots/cobots], this article uses the term “robots” to refer to “cobots”—collaborative robots (new 4.0 technology that “assist the human worker in conducting a common task”) and cooperative robots (new 4.0 technology that “work individually in the vicinity of the worker”) (Weckenborg et al., 2022).

Inside the Industry 4.0 organizational systems, the human can be a vulnerable element (Nuamah and Seong, 2017), an element that may be treated superficially or may even be ignored. Therefore, in balance with the economic approach of the organizational systems is needed a sociotechnical approach specific to human factors/ergonomics (HF/E) (Dul et al., 2012; Neumann et al., 2021). More specifically, the attention to humans inside the organization should be integrated with the engineering design and management process to obtain a sustainable competitive advantage (Neumann et al., 2021).

The new concept Industry 5.0 complements the technology-oriented Industry 4.0 with “a sustainable, human-centric, and resilient focus” (Oztemel and Gursev, 2020; Breque et al., 2021 apud Reiman et al., 2021). The use of an Industry 5.0 approach, associated with taking into consideration not only processes’ automation to make them more efficient but also the human costs resulting from process optimization (Nahavandi, 2019), is mandatory for organizational sustainability.

This brief research report explores the new technologies’ impact, especially robots, on the company and on employees’ wellbeing and spiritual fulfillment. As far as we know, aspects related to wellbeing at work were studied before but there is a gap in research related to the spiritual approach of the human inside the organization for performance enhancement. The research results consist of two managerial instruments for human capital valorization (HCV) inside organizations, especially those which intend to invest in robots and new 4.0 technologies.

This article is structured in five sections. After the introduction, the main coordinates of the research methodology and the research objectives are presented. The third section draws an overview of the research background. The fourth section presents the results of the interviews taken to explore today’s challenges and the new 4.0 technologies’ impact, especially robots, on the company and on employees’ wellbeing and spiritual fulfillment. The fifth part presents the managerial instruments developed and validated within the qualitative research, points out the novelty and original elements, the limitations, and further directions for study. The last part highlights the main conclusion.

## Research Methodology

The next part presents the main coordinates of the research methodology: choosing the research team, defining the research problem and research objectives, designing the interviews questions, participants, and outcomes.

The research methodology followed six stages. This brief research report presents, in the next parts, the outputs and outcomes of two intermediary research stages (Stage 2 and Stage 5) related to developing, testing, and validating the research model. Stages 1, 2 and 3 of the study were part of research supported between July 2019 and July 2020 by the *ValoRes* project (titled “Research regarding human capital’s influence on organization’s market performance and practical recommendations,” a project with an economic environment that aimed to explore new ways of analysis and diagnosis of the human capital influence on organizational results). Stages 4, 5 and 6 of the study were not funded by grants or research projects.

The research team was coordinated by an HF/E specialist (the first author of this article), and it consisted of experts in the fields of: integrated management systems (IMS) (the second author), psychology and theology (the third author), and business growth (the fourth author). The research process planning was done during the team’s first meeting when it was decided upon: the expected results, analysis criteria, necessary resources, time frame, interviewing methods and protocol, etc.

### Stage 1—Literature Review for Defining Research Objectives and Framework

In *Stage 1* of our research, a literature review was undertaken to find “what is known” about the main question of our research: *How can companies improve their market performance using their human capital?* The review objectives were: O1—To map the connection between market performance, organizational performance, and human capital performance, O2—To understand the new challenges in organizations and human capital management.

To achieve the research goals, a literature search was performed in January 2020 (Stage 1) and was supplemented with updated articles in September 2020 (Stage 4) and February 2022 (Stage 6).

In addition to two books (Huselid et al., 2005; Mayo, 2014), 35 articles were identified using the Scopus, Wiley Online Library, Taylor & Francis Online, APA Psyc Net, IEEE Xplore, and Pub Med databases. The search focused on articles title and abstract, in English, without time limitation, sorted by relevance and using a combination of keywords. To identify the articles focusing on the research interest area, the literature relevance was assessed based on the title and abstract.

To achieve O1, in the first step of Stage 1, the search used the keywords: “market performance,” “human capital performance,” “work performance,” “human capital,” “workforce,” “human resources,” and “talent management.” In this step, 15 selected articles (Harel and Tzafrir, 1999; Beatty et al., 2003; Schneider et al., 2003; Combs et al., 2006; Crook et al., 2011; Kamya, 2012; Harpan and Draghici, 2014; Folds, 2015; Rasmussen and Ulrich, 2015; Hecklau et al., 2016; Marler and Boudreau, 2017; Huselid, 2018; Minbaeva, 2018; Whysall et al., 2019; Pagán-Castaño et al., 2020) were read in full to map the connection between market performance, organizational performance, and human capital performance.

To achieve O2, in the second step of Stage 1 the search used, based on the results obtained in the previous step, the keywords: “Industry 4.0,” “robots,” “new 4.0 technologies,” “sustainability,” “sociotechnical systems,” and “macroergonomics.” To understand the new challenges in organizations and human capital management, 20 articles were selected and read in full: 10 articles (Kinzel, 2016; Bertacchini et al., 2017; Bortolini et al., 2017; Gašová et al., 2017; Nuamah and Seong, 2017; Mikulic and Stefanic, 2018; Kadir et al., 2019; Nahavandi, 2019; Sanders et al., 2019; Ghobakhloo, 2020) identified using a combination of “Industry 4.0/robots/new technologies” and “ergonomics/sustainability” keywords, 6 articles (Kleiner, 2006; Dul and Ceylan, 2011; Dul et al., 2012; Karltun et al., 2017; Realyvásquez-Vargas et al., 2018; Greig et al., 2019) identified using “macroergonomics/organizational ergonomics” keywords, and 4 articles (Bolis et al., 2014; Zink, 2014; Radjiyev et al., 2015; Thatcher and Yeow, 2016) identified using a combination of “sustainability” and “ergonomics” keywords.

The *Stage 1* outputs let to two secondary research questions, as follows:

*SQ1: How is the future foreseen about robots’ use in sustainable organizations, from the systemic and spiritual perspective?*

*SQ2: Which are the challenges that occur in organizations for HCV, in the context of using new 4.0 technologies, especially robots?*

Furthermore, three secondary research objectives were set, as follows:

SO1—To understand the interactions between personal, technological, and organizational subsystems, in the context of robots use in organizations.

SO2—To understand the new challenges regarding new 4.0 technologies’ impact, especially robots, on the company and on employees’ wellbeing and spiritual fulfillment.

SO3—To shape a model for human capital’s valorization and to suggest a list for monitoring HCV in organizations, in the context of using new 4.0 technologies, especially robots.

Based on the outputs of the multidisciplinary bibliographic study performed, the HF/E specialist drafted a theoretical research model, which was the basis for the interviews with three experts in *Stage 2* and *Stage 5* of our research.

### Stage 2—First Round of Interviews for Testing and Developing the Research Model

The research model testing and development was done in *Stage 2* of our research, through two one-to-one semi-structured interviews. The main themes of the discussions focused on today’s managerial challenges and new technologies’ impact, in particular robots, on employees’ wellbeing, spiritual fulfillment, motivation, and productivity. The data registered during the interviews were written down, summarized, discussed, and later reviewed by the interviewees.

The first round of interviews (March 2020) was performed by the HF/E specialist, using videoconference facilities. The length of the interviews was about 80 min (interview 1) and 60 min (interview 2), with a medium length of about 70 min.

*Interview 1* aimed to highlight the essential aspects related to the contribution brought by IMS for the successful use of robots in organizations. The participant was a Romanian woman, age group 45–49, engineer, with more than 20 years of experience in implementation, audit, and consultancy in the field of IMS. The interview 1 discussions followed the focal question *“How is future foreseen about robots’ use in sustainable organizations, from the systemic perspective?”* and were supported by the following questions:

*I1-Q1: Should organizations be prepared for robots use?*

*I1-Q2: What part do leadership and IMS play for sustainable robots use in organizations?*

*I1-Q3: What part does ergonomics play in the trinomial robots use—IMS—sustainability?*

*I1-Q4: How will new technologies, especially robots, influence organizations from an IMS perspective?*

*Interview 2* aimed to highlight the essential aspects related to the employee’s adjustment to the use of robots in organizations. The participant was a Romanian man, age group 45–49, theologist having intercultural psychological studies, with more than 20 years of experience in human knowledge and development. The interview 2 discussions followed the focal question “How is future foreseen about robots use, from the spiritual perspective?” and were supported by the following questions:

*I2-Q1: Should organizations be prepared for robots use?*

*I2-Q2: How should employees prepare for robots use in organizations?*

*I2-Q3: What challenges occur for employees and organizations, from the spiritual perspective?*

The main outcome of the first round of interviews consisted in testing and development of the HCV model, presented in this brief research report.

### Stage 3—Quantitative Research for Developing the Research Framework

The outputs of the first round of interviews were used in *Stage 3* of our research, with the aim to identify Romanians’ interest in using telework and new technologies in organizations for performance enhancement (May 2020).

A quantitative, not random research based on a questionnaire was performed on a sample of 363 Romanian respondents (Firescu et al., 2020; Firescu and Gaşpar, 2020). The term “new technologies” was explained in the questionnaire as follows: collaborative robots, devices equipped with smart sensors, remotely managed technologies and devices, artificial intelligence—technologies based on voice and biometric recognition. The main results highlighted respondents’ opinions regarding possible barriers for implementing new technologies: (1) financial reasons (high costs), (2) educational reasons (lack of information, lack of instructions for technology use, and lack of training), and (3) emotional reasons (fear of change, fear of making mistakes, and fear of being monitored). The results also highlighted a list of continuing education programs related to performance enhancement: technological novelty, ergonomics (managing human-technology relationship), and vitality management (work-life balance, health and vitality, and overwork avoidance).

### Stage 4—Literature Review for Developing the Research Model

Based on *Stage 2* and *Stage 3* outputs, in *Stage 4* of our research, the literature review was supplemented with updated articles searched on the internet (September 2020), using combinations of the keywords: “Industry 4.0/ergonomics” and “sustainability/wellbeing/work-life balance/emotion.” Based on the same criteria mentioned above (Stage 1), 7 articles (Zapf et al., 2010; Hofmann and Stokburger-Sauer, 2017; Fotiadis et al., 2019; Bolis et al., 2020; Brunoro et al., 2020; Gualtieri et al., 2020; Oztemel and Gursev, 2020) were selected and read in full. This stage aimed to find scientific arguments for all the elements included in the HCV model.

### Stage 5—Second Round of Interviews for Developing and Validating the Research Model

In *Stage 5* of our research, the validation of the HCV model was done through *Interview 3*, organized in two parts. The participant was a Romanian man, age group 55–59, a doctor with over 25 years of experience in management, training, coaching, and consultancy for business consolidation and growth, with practical experience in hundreds of companies. The interview was performed by the HF/F specialist, at the participant’s office.

*Interview 3—Part 1* (October 2020) has a length of about 50 min and aimed to develop the HCV model from the business strategic perspective. The discussions followed the focal question *“Which are the challenges that occur in organizations in the context of HCV?”* and were supported by the following questions:

*I3-Q1: Which is the business consultant’s opinion about the research model, from its practical experience in hundreds of companies?*

*I3-Q2: What challenges arise in organizations for valorizing the human capital?*

*Interview 3—Part 2* (January 2021) has a length of about 40 min and aimed to validate the HCV model and HCV monitoring list presented in this article. The discussions followed the focal question *“Which is the business consultant’s opinion about the results regarding organizational ergonomics’ contribution to employees’ wellbeing and organizational performance, in the context of using new 4.0 technologies, especially robots?”* and were supported by the following questions:

*I3-Q3: Which is the business consultant’s opinion about the HCV model utility, from its practical experience in hundreds of companies?*

*I3-Q4: Which is the business consultant’s opinion about the HCV monitoring list utility, from its practical experience in hundreds of companies?*

The *Stage 5* outcomes consisted in validating the HCV model and HCV monitoring list, from the business perspective.

### Stage 6—Literature Review for Analyzing the Research Model Utility

To validate the HCV model and monitoring list elements and utility from the research and scientific perspective, following the main question of our research “*How can companies improve their market performance using their human capital?*,” in *Stage 6* of our research the literature review was supplemented with updated articles search on Scopus database (January and February 2022). A total of 11 articles were selected and read in full, based on the same criteria mentioned above *(Stage 1)*: 4 articles (Waldeck et al., 2021; Metz et al., 2022; Samson and Bhanugopan, 2022) identified using “human capital,” “human capital analytics,” and “human resources analytics” keywords, 2 articles (Chiang et al., 2020; Wixwat and Saucier, 2021) identified using “spiritual” keyword, and 6 articles (Neumann et al., 2021; Reiman et al., 2021; Segura et al., 2021; Baber and Young, 2022; Ching et al., 2022; Weckenborg et al., 2022) identified using “Industry 4.0,” “robots,” “ergonomics,” and “sustainability” keywords.

The *Stage 6* outputs were used for arguing the utility of the management instruments developed for supporting decision-makers and human capital analysts.

## Research Background

In the last years, many researchers focused on integrating Industry 4.0, sustainability, and ergonomics to highlight the positive impact of Industry 4.0 on sustainable development (Ghobakhloo, 2020; Ching et al., 2022), to identify solutions for improving companies’ decision-making processes to increase employee’s wellbeing (Bolis et al., 2020) and for framing the elements that lead to organizational competitive advantage (Reiman et al., 2021).

The discussion about Industry 4.0 (Ghobakhloo, 2020; Oztemel and Gursev, 2020; Reiman et al., 2021; Ching et al., 2022) regards its technology-oriented approach, focusing especially on increasing systems productivity and flexibility and less on the human-centric, sustainable, and resilient approach.

The new concept of Industry 5.0 brings into discussion the sociotechnical approach specific to HF/E (Dul et al., 2012; Neumann et al., 2021) in balance with the economic approach of the organizational systems. However, companies’ management aims at the economic criteria in the decision-making process (Weckenborg et al., 2022), and research is needed for arguing the need for an Industry 5.0 sustainable approach. As Samson and Bhanugopan (2022) mentioned, managerial decision-making determines organizational performance (product quality, employee attraction and retention, customer satisfaction, etc.) and influences market performance (sales, market share, company profitability, etc.).

Going further on the issue of increasing market and organizational performance, it is necessary for organizations to connect learning activities objectives with information regarding the external environment, market, and customers’ needs (Kamya, 2012). Previous studies have recommended the implementation of human resources management (HRM) practices and activities to connect employees’ abilities with organizational strategy and to increase employees’ motivation, adaptability, and flexibility (Waldeck et al., 2021). Employee’s adaptability promotes wellbeing (Waldeck et al., 2021), and HRM practices oriented to wellbeing have a positive impact on employees (Folds, 2015; Pagán-Castaño et al., 2020).

Going further on the issue of human resources/human capital management, Whysall et al. (2019) pointed out the need for changes in strategic HRM, by using a talent management approach of humans at work in the context of new 4.0 technologies use, and going toward a more dynamic systemic thinking, to allow organizations to be competitive. Furthermore, Zhang and Chen (2021) mentioned also the need for “talent education curriculum” design and planning, for talents training “in a high-tech industry.”

The next concern on the research topic covers ergonomics’ impact on organizational performance. As mentioned by Realyvásquez-Vargas et al. (2018), macroergonomics (part of HF/E) impacts organizational performance by reducing production errors, decreasing OH&S risks at the workplace, and improving employees’ satisfaction and quality of life.

Sustainable companies’ investments in ergonomics are linked especially to occupational disease control, occupational risks prevention, and designing healthy working environments, and impacts also the organization’s social performance (e.g., the ergonomic biophilic workplaces’ design allows employees wellbeing, and the work systems’ ergonomic design allows gender diversity) (Firescu, 2021; Neumann et al., 2021).

The multidisciplinary bibliographic study performed in step 2 of Stage 1, of the research methodology, regarded the impact of new technologies implementation in organizations (Mikulic and Stefanic, 2018; Kadir et al., 2019; Whysall et al., 2019) and highlighted the HTO concept perspective on the Human-Technology-Organization (HTO) relationship, suggested by Karltun et al. (2017).

Starting from the work system components, analyzed from a macroergonomic view (Kleiner, 2006; Zink, 2014; Thatcher and Yeow, 2016), the theoretical research model was defined by adding the HRM’s strategic perspective, through the analysis of challenges brought in by talents’ management (Whysall et al., 2019), and the human value analysis in organizations (Huselid et al., 2005; Mayo, 2014). Furthermore, the emotional labor perspective was added, through the analysis of challenges brought in by emotions management (Zapf et al., 2010) and their impact on employees’ work-life balance and wellbeing (Hofmann and Stokburger-Sauer, 2017; Fotiadis et al., 2019).

Considering the integrated approach presented above, the HF/E specialist suggested a theoretical research model, which was the basis for the interviews with three experts. The testing and development of the research model was done through three one-to-one semi-structured interviews, focused on the focal questions presented in the previous section of this article. The main themes of the discussions focused on today’s managerial challenges and new technologies’ impact, in particular robots, on employees’ wellbeing, spiritual fulfillment, motivation, and productivity.

## Results

**FIGURE 1:**
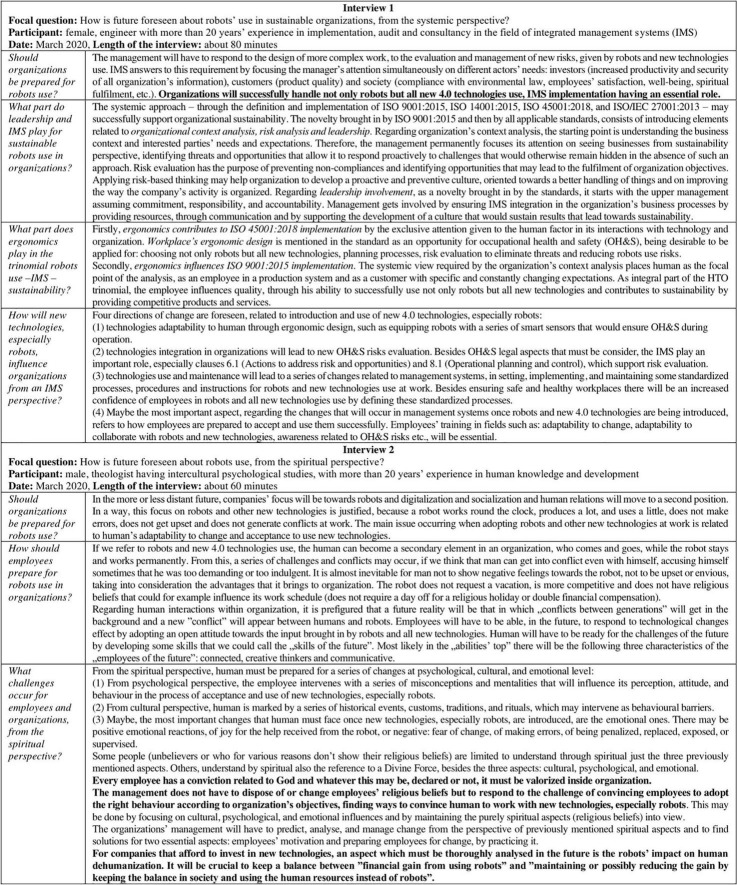
Outputs of the first round of interviews.

**FIGURE 2:**
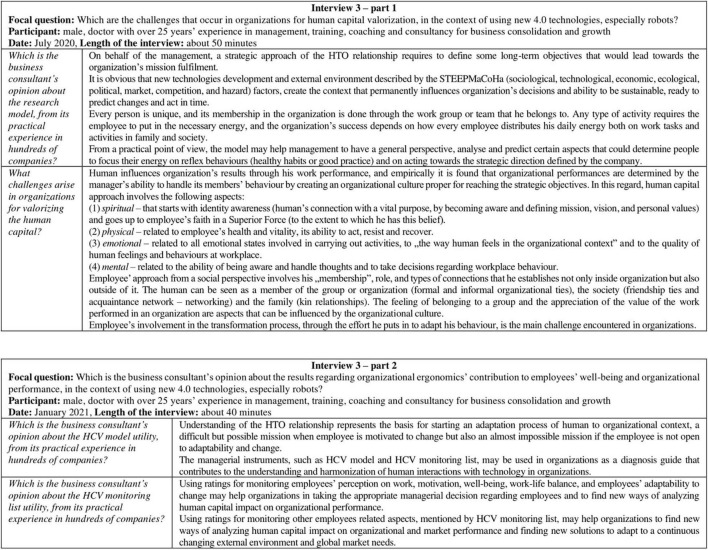
Outputs of the second round of interviews.

The next part of the article presents the research results and is based on the data registered during the two rounds of interviews. The results were structured according to the answers, were reviewed by the interviewees, and were summarized following the interviews questions (Figures 1, 2).

As presented in the research methodology section, interview 1, interview 2, and interview 3—part 1 were performed for testing and development of the research model. For the validation of the managerial instruments was performed part 2 of interview 3. The interviews’ results are presented separately in this section and will be discussed integrated with the next part of the article.

During *Stage 4* and *Stage 6* of our research, the data registered during the interviews were checked to validate the HCV model and monitoring list elements from the research and scientific perspective. For example, it was found that spirituality is described differently in non-Europe-Christian-centered cultures and Europe-Christian-centered cultures (Wixwat and Saucier, 2021). For the later ones, spirituality is described as “a personal, subjective experience” related to tradition, culture, community, and identity elements (Wong and Vinsky, 2009 apud Wixwat and Saucier, 2021). Likewise, spirituality is associated with the cultural background and religious beliefs, meaning a person’s experiences and beliefs, connections with the self, others, nature, and God, for providing harmony and meaning in human lives (Rothman, 2009; Barber, 2012 apud Chiang et al., 2020; Timmins and Neill, 2013).

Regarding possible negative feelings of humans toward the robot (being upset or envious), mentioned by interview 2 participant, it was found that frustration was identified at the maintenance staff for a picking cobot (collaborative robot) and at the IT staff for augmented reality for machine maintenance (Neumann et al., 2021).

## Discussion

The next part of the article presents the research contributions, discussing the key elements of the managerial instruments presented in this article, the novelty and original elements of this research, the limitations and future directions for study and exploration.

### The Key Elements of the Human Capital Valorization Managerial Instruments

**FIGURE 3:**
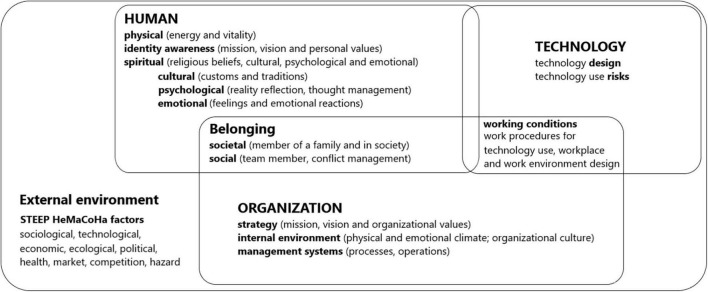
The human capital valorization (HCV) model.

**FIGURE 4:**
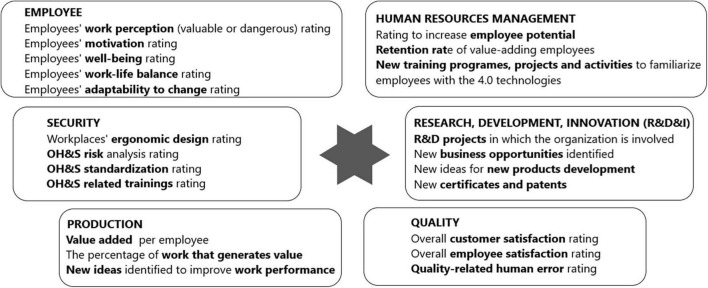
The human capital valorization (HCV) monitoring list.

The results of the qualitative research were integrated with two managerial instruments (the HCV model, Figure 3 and the HCV monitoring list, Figure 4), tested and validated from the business perspective.

#### The Human Capital Valorization Model Key Elements

Although the new 4.0 technologies like robots, automation and assistance technologies are more and more used and continuously developed, the role of a human in still essential in the organization, especially in operations systems (Neumann et al., 2021; Reiman et al., 2021). Considering the need for new ergonomic approaches in the context of new technologies use (Neumann et al., 2021; Reiman et al., 2021; Baber and Young, 2022), the HCV model was developed to help managers to understand HTO interactions from a sociotechnical perspective, a macroergonomics one, and to predict certain aspects that could determine employees to act toward the company strategic direction.

The HCV model includes three key elements: Human (H), Technology (T), and Organization (O), and three key connections elements: the Belonging, the Working conditions, and the External environment.

The first key element, the Human, is defined by a person’s identity awareness (employee mission, vision, and personal values), physical aspect (person physical energy and vitality), and spiritual aspect (including the religious beliefs together with psychological, cultural, and emotional aspects). The Human interactions with the other key elements will impact the work motivation and performance as well the work value perception, feeling included or excluded, valuable or less valuable within the organization.

The second key element, the Organization, is defined by strategic management system (including company mission, vision, and organizational culture), management systems (including processes and operations), and internal work environment (physical and psychological climate). This key element together with the key connection element External environment will influence the management perception of the employee’s value inside the organization.

The third key element, the Technology, is defined by human-centered technology design and technology OH&S use risks. This key element, together with the key connection element Working conditions will influence employees’ health and wellbeing at work.

The Belonging, the first key connection element, takes into consideration social aspect (employee organizational membership) and societal aspect (employee membership in the community). The feeling of belonging to a group and the appreciation of the value of the work performed in an organization are aspects that can be influenced by the organizational culture.

The Working conditions, the second key connection element, refer to the workplace and work environment design, development of specific work procedures, and performing of new OH&S risks analysis for use of new technologies.

The External environment, the third key connection element, described by the STEEP HeMaCoHa factors, creates the context that permanently influences an organization’s decisions and ability to be ready to predict changes and to act sustainably in complex situations. Health factors have been explicitly specified in the model, taking into consideration the worldwide experience of the 2020 COVID-19 pandemic and its impact on organizations functioning.

#### The Human Capital Valorization Monitoring List Key Elements

To be competitive, organizations should permanently monitor the added value brought in by every employee, every project (such as implementing new 4.0 technologies), and every customer, and use the monitoring results in the managerial decision-making process. The HCV monitoring list focuses on six aspects: employee, security, production, quality, HRM, and R&D&I (research, development, and innovation). The list presents examples of KPIs related to the six key elements.

Using ratings and KPIs for monitoring employees’ perception of work, motivation, wellbeing, work-life balance, and employees’ adaptability to change may help organizations in taking the appropriate managerial decisions regarding employees and to find new ways of analyzing the human capital impact on organizational performance.

Using ratings and KPIs for monitoring security, production, quality, HRM, and R&D&I may help organizations to make a diagnosis of human influences on the social and economic organizational performance, to find new ways of analyzing the human capital impact on organizational and market performance and to find new solutions to adapt to a continuous changing external environment and global market needs.

### Novelty and Original Elements of the Human Capital Valorization Model and Monitoring List

This section highlights the novel and original elements of the research and shows arguments for considering the organizational ergonomics’ contribution to employees’ wellbeing (Dul et al., 2012; Neumann et al., 2021; Baber and Young, 2022).

#### Connecting Science and Business Views

The managerial instruments presented in this article were validated using a methodology that allowed a correlated perspective between scientific and business views.

The framework of the research provides a systemic, emotional, and spiritual approach to man’s interaction with technology, systems, and organizational environment, and is based on sociotechnical macroergonomics perspective (Zink, 2014; Karltun et al., 2017; Mikulic and Stefanic, 2018). Understanding the HTO relationship may increase the manager’s awareness of important aspects related to the employee’s personality and its influence on work performance and help managers to find new innovative ways for harmonizing the HTO interaction.

Regarding the utility of the managerial instruments presented in this article, the HCV model and HCV monitoring list may be used in organizations as a diagnosis guide that contributes to the understanding and harmonization of human interactions with new technologies in organizations. The diagnosis of the HTO relationship represents the basis for starting the adaptation process of human to the organizational context, to increase organizational performance (Waldeck et al., 2021), but also the basis for finding new inputs in the human capital analytics process (Samson and Bhanugopan, 2022).

#### Including the Spiritual Aspects in Human Factor Approach

The main novelty element of the HCV model regards the focus on the spiritual aspects in the human factor approach within an organization. To increase the value-added to the organization by the human capital, a differentiated approach of the employees will be absolutely necessary, by taking into consideration employee identity, physical, cultural, emotional, psychological, and spiritual aspects.

As the research results showed, every employee has a conviction related to God and whatever this may be, declared or not, it must be valorized inside the organization. The management does not have to dispose of or change employees’ religious beliefs but to respond to the challenge of convincing employees to adopt the right behavior according to the organization’s objectives. For managing the future challenges raised by a “possible conflict” between humans and robots, one could say that the manager will have to mediate between human and robot to increase their collaboration, and to be as Saint Apostle Paul: able to formulate messages and to use differentiated approach strategies, suitable for every employee (Crucianu, 2010).

The HCV model claims that the management strategies have to be focused on employees’ identity, personality, needs, and expectations. This may be done by focusing on cultural, psychological, and emotional influences and by maintaining the purely spiritual aspects (religious beliefs) into view.

#### Including the Belonging, as a Key Connection Element

Besides the H key element aspects included in the HCV model, the human can be seen as a member of the group or organization (formal and informal organizational ties), member of the society (friendship ties and acquaintance network—networking), and member of the family (kin relationships).

An original element of the model refers to the inclusion of the Belonging key connection element, to consider the employee’s feeling of belonging to the organization (as a team member), and to the community (as a family and society member). The way the employee perceives his belonging to an organization influences his work motivation and the orientation of his energy and behavior toward obtaining organizational performance.

### Limitations and Future Directions

As presented in the research methodology section, the interviews discussions focused especially on collaborative robots (Oztemel and Gursev, 2020; Segura et al., 2021; Weckenborg et al., 2022) use in organizations. Considering this aspect, the results can be used for analyzing the collaboration between humans and robots in organizations, with a focus on industry. Further validation is needed before using the instruments presented in this article for improving the employee’s adaptability to change and collaboration with other new 4.0 technologies (such as cooperative robots, IoT systems, cloud-based systems, and artificial intelligence).

Considering the Industry 5.0 approach based on sustainability, human-centric design, and resilience (Nahavandi, 2019; Reiman et al., 2021), the need for collaboration between engineering, HF/E, and business practice is mandatory. As was mentioned, the research model was tested, developed, and validated based on three interviews performed by an HF/E specialist. Even all participants have more than 20 years of experience in business growth, human knowledge and development, and implementation, audit, and consultancy in IMS, all participants were Romanian specialists. It must be mentioned that the research results may present a restricted perspective, with cultural influences.

Regarding further directions of the research, the challenges pointed out in this section regard (1) identifying and developing managerial solutions (tools and instruments) for a smooth integration of the human factor in the IoT systems, using HF/E and Industry 5.0 approach, (2) quantifying the costs and value of human factor in organizational context, (3) exploring directions of collaboration between HF/E and HCA/HRA (human capital/human resources analytics) (Bolis et al., 2020) with a double aim: making ergonomics accountable and making employee’s identity, personality, needs, and expectations countable from business strategic perspective, and (4) analyzing the impact of new technologies use on human being and exploring solutions for a sustainable use of new technologies, to prevent a potential human dehumanization (for example, benefits for companies, to maintaining the balance between “financial gain from using robots” and “maintaining or possibly reducing the gain by keeping the balance in society and using human resources instead of robots”).

## Conclusion

Starting from the main question of the study *“How can companies improve their market performance using their human capital?,”* the qualitative research presented in this article focused on an integrated systemic—emotional—spiritual approach of human capital.

The practical contribution of the research consists in developing two managerial instruments, to help managers easily understand HTO interactions and to predict aspects that could determine employees to act toward the company strategic direction. The instruments were validated based on three one-to-one interviews performed by an HF/E specialist, with Romanian specialists having more than 20 years of experience in business growth, human knowledge and development, and IMS. The research aimed to validate a model for human capital’s valorization and to suggest a list for monitoring HCV inside organizations, in the context of robots and new 4.0 technologies use. Although the research had the limitations presented in the previous section, the first research results are promising and bring into attention the importance of the Industry 5.0 revolution.

## Data Availability Statement

The original contributions presented in the study are included in the article/supplementary material, further inquiries can be directed to the corresponding author.

## Author Contributions

VF: writing original draft, literature review, and methodology. VF, M-LG, IC, and ER: deeper data analysis, conceptualization, and validation through research and investigation. All authors have read and agreed to the final version of the manuscript.

## Conflict of Interest

ER was employed by De KLAUSEN. The remaining authors declare that the research was conducted in the absence of any commercial or financial relationships that could be construed as a potential conflict of interest.

## Publisher’s Note

All claims expressed in this article are solely those of the authors and do not necessarily represent those of their affiliated organizations, or those of the publisher, the editors and the reviewers. Any product that may be evaluated in this article, or claim that may be made by its manufacturer, is not guaranteed or endorsed by the publisher.
